# Detecting Unseen IoT Attacks with Calibrated Dual Evidence Under Low False-Positive Budget

**DOI:** 10.3390/e28091026

**Published:** 2026-09-15

**Authors:** Jiahui Yue, Yuliang Lu, Yi Xie

**Affiliations:** 1 College of Electronic Engineering, National University of Defense Technology, Hefei 230037, China; audreyue@163.com (J.Y.); luyuliang@nudt.edu.cn (Y.L.); 2 Anhui Province Key Laboratory of Cyberspace Security Situation Awareness and Evaluation, Hefei 230037, China

**Keywords:** Internet of Things, network traffic anomaly detection, unseen-attack detection, empirical score calibration, benign-tail surprisal, false-positive rate control

## Abstract

Internet of Things (IoT) traffic anomaly detection is essential for limiting device compromise and large-scale attacks. Existing detectors may miss attack families absent from model development, while heterogeneous benign traffic makes it difficult to maintain a low false-positive rate (FPR). To address these two practical limitations, we propose the Mode-Calibrated Dual-Evidence Detector (MCDE). Its supervised branch estimates the probability that a sample is malicious from labeled benign and known-attack traffic, while its benign-deviation branch measures distance from multiple learned benign traffic modes, providing a complementary route for unseen attacks. MCDE maps the heterogeneous probability and distance scores to comparable empirical benign-tail evidence, normalizes each branch by its allocated share of the target FPR, and fuses them into an anomaly score. A disjoint held-out benign set determines the decision threshold. Equivalently, the fusion compares budget-adjusted benign-tail surprisal, linking the decision rule to empirical self-information. We further establish the conditions under which the budgeted fusion controls the nominal overall FPR. Family-hold-out experiments on IoT-23 and N-BaIoT validate MCDE. At a 1% target benign FPR, MCDE improves IoT-23 unseen recall over histogram-based gradient boosting from 85.77% to 90.85% and harmonic known–unseen recall from 91.82% to 95.07%, while maintaining a 0.96% benign-test FPR. It also achieves 99.87% unseen recall on N-BaIoT.

## 1. Introduction

The rapid deployment of Internet of Things (IoT) devices has expanded the attack surface of modern networks [1,2]. Their constrained resources, long service lifetimes, and heterogeneous protocols expose IoT systems to weak authentication, insecure communication, vulnerable software, privacy leakage, denial-of-service attacks, and botnet propagation [3,4,5]. These threats can compromise sensitive data, disrupt service availability, and facilitate attacks against interconnected devices and physical processes, making IoT security an important ecosystem-level concern [6,7]. IoT traffic anomaly detection therefore provides an important layer of defense by analyzing statistical patterns in network traffic data to distinguish suspicious activity from legitimate device communication, without modifying individual devices or inspecting packet payloads [8,9].

Existing IoT traffic anomaly detection methods employ machine-learning and deep-learning models under supervised, semi-supervised, and unsupervised settings [10,11]. Representative studies have reported strong discrimination between benign and malicious traffic under controlled benchmarks [7,12,13]. However, many studies focus on attack patterns represented during model development and do not explicitly consider unseen attack families, whose samples are entirely absent from the development process. This capability is important in practice because new or evolving attacks may appear before representative samples and labels can be collected.

Several approaches have attempted to address incomplete attack knowledge. Benign-only normality models can detect deviations without requiring attack examples, but they cannot exploit known-attack labels and may miss attacks that remain close to learned benign behavior [14,15]. Open-set and hybrid detectors combine known-attack information with benign structure, but their heterogeneous outputs still require a principled decision mechanism [16]. Online learning and few-shot adaptation can incorporate emerging attacks, but require target-family samples, labels, feedback, or model updates [17,18]. Moreover, family-hold-out studies show that strong performance on represented attacks does not necessarily imply reliable detection of a completely unseen family [19]. The remaining challenge is therefore to preserve the discrimination learned from known attacks while providing a complementary detection route for such unseen attacks.

Beyond unseen-attack sensitivity, a low false-positive rate (FPR) is equally important for practical deployment. Since benign traffic greatly outnumbers malicious traffic in routine IoT monitoring, even a small FPR can generate a substantial alarm burden [8,20]. For a given detector, relaxing the decision criterion may improve unseen-attack recall by enlarging the range of traffic considered suspicious, but it admits more benign samples into the alarm set. This trade-off is particularly relevant in heterogeneous IoT environments, where different devices, services, operating states, and capture conditions may produce distinct legitimate traffic patterns [13,21,22]. Practical unseen-family detection must therefore improve sensitivity beyond known attack patterns without obtaining such gains through excessive false alarms.

Motivated by these requirements, we propose the Mode-Calibrated Dual-Evidence Detector (MCDE), a decision framework for unseen-family detection at a predefined low-FPR operating point. MCDE designs two branches to produce complementary raw scores. The supervised branch outputs an estimated malicious-class probability learned from labeled benign and known-attack traffic, whereas the benign-deviation branch computes a mode-conditioned distance that quantifies deviation from learned benign traffic behavior. Since the two scores have different semantics and distributions, each is ranked against its branch-specific benign calibration reference. This converts them into empirical benign-tail evidence on a common scale, where a larger value indicates greater extremeness relative to benign traffic. MCDE then assigns each evidence source a share of the target FPR and fuses the budget-adjusted evidence into a single anomaly score. A second disjoint benign set calibrates the decision threshold for this fused score, yielding the final benign-or-malicious classification. Through this design, MCDE combines complementary sensitivity to unseen attacks with operation at a prescribed low-FPR point.

Accordingly, the main objective of this study is to develop and evaluate a family-disjoint IoT traffic anomaly detector that preserves discrimination for known attacks, provides complementary sensitivity to attack families unavailable throughout model development, and operates under a predefined low-FPR constraint. The main contributions of this study are summarized as follows:

- Mode-calibrated dual-evidence detection. We propose MCDE, which combines supervised evidence for known attacks with local-deviation evidence derived from multiple benign traffic modes. The two branches have distinct responsibilities. The supervised branch preserves discrimination for known attack families, whereas the benign-deviation branch provides complementary sensitivity to unseen samples that are poorly represented by heterogeneous benign behavior.
- Benign-tail alignment and FPR-budgeted decision calibration. We develop a non-parametric mechanism that maps heterogeneous probability and distance scores to a common empirical benign-tail scale and fuses them under branch-wise shares of a nominal FPR budget. Using a union-bound argument, we establish conditions under which the combined nominal benign-alarm probability remains within the requested budget without assuming branch independence. We further express the budget-normalized fusion through the negative logarithm of each calibrated benign-tail probability, using empirical self-information to interpret how the FPR shares determine the evidence required from each branch.
- Comprehensive low-FPR unseen-family evaluation. We evaluate MCDE through family-disjoint hold-outs, group-wise FPR analysis, stress tests, ablations, sensitivity studies, paired analysis, and fair CPU-only benchmarking on IoT-23 and N-BaIoT. At a 1% target benign FPR, MCDE raises IoT-23 unseen recall from 85.77% to 90.85% and harmonic known–unseen recall from 91.82% to 95.07% relative to histogram-based gradient boosting (HGB) while maintaining a 0.96% benign-test FPR. It also achieves 99.87% unseen recall on N-BaIoT.

The remainder of this paper is organized as follows. Section 2 reviews existing IoT traffic anomaly detection methods. Section 3 presents the MCDE framework and its calibration procedure. Section 4 describes the experimental design, reports the results, and analyzes their scope and limitations. Section 5 concludes the paper.

## 2. Related Work

Existing IoT traffic anomaly detection methods can be broadly grouped according to how they use attack information. We first consider methods trained with labeled known attacks, then methods that use limited labels or adapt to newly available information, and finally methods that learn without attack labels. The following subsections review these three technical routes.

### 2.1. Detection from Labeled Known Attacks

Supervised IoT traffic detectors learn a direct mapping from traffic-derived features to benign and malicious labels. Conventional machine-learning models remain common in this setting because tree ensembles and gradient-boosting methods handle nonlinear interactions in tabular traffic statistics with moderate computational cost. Tyagi and Kumar [10] compared supervised learning approaches for IoT attack detection, while De Souza et al. [23] used an Extra Trees detector followed by an ensemble that identifies intrusive traffic. Mishra et al. [24] optimized a LightGBM detector for malicious IoT access, illustrating the continued effectiveness of boosted trees on structured network features. Farrukh et al. [16] extended the supervised ensemble route to open-set detection by clustering benign traffic and combining subgroup-specific base learners through meta-classifiers trained on benign and known-attack samples. Such models provide strong and reproducible baselines when representative attack labels are available.

Deep supervised models seek more expressive representations or decision functions from the same labeled setting. Vector-convolutional models, recurrent networks, and multitask architectures have been used to detect malicious traffic or identify the corresponding attack or malware type [9,25]. Zhao et al. [11] combined decision-tree-based feature processing with a proximal policy optimization detector, using information entropy for feature selection and entropy regularization in the learning objective. Comparative evidence nevertheless indicates that robustness depends on preprocessing, dataset construction, and evaluation design as well as architecture depth [13].

Conventional supervised classifiers exploit known-attack labels to learn discriminative malicious regions. However, their unseen-family performance depends on whether the evaluation excludes target families rather than on model depth alone. Random or stratified record-level splits often retain the same attack families in training and testing, whereas family-hold-out evaluations show that both shallow and deep classifiers can lose recall on attacks excluded from model development [19]. Therefore, increasing model complexity alone does not overcome incomplete attack knowledge, motivating methods that use limited supervision or adapt to newly available information.

### 2.2. Detection with Limited or Incrementally Acquired Attack Information

Semi-supervised methods use partial attack supervision while retaining an anomaly-detection objective. For example, Deep SAD [26] extended deep one-class classification by incorporating a small number of labeled anomalies into representation learning. When a few labeled samples from target attacks are available, few-shot learning provides another option. Bovenzi et al. [27] evaluated metric- and representation-learning designs for IoT attack-traffic classification with limited labeled examples and reported improvements over non-few-shot baselines.

Other methods use information acquired from deployment or a target domain. Wu et al. [17] proposed an adaptive online ensemble for concept drift and class imbalance in IoT streams. Saleem and Hamouda [18] combined federated and few-shot learning with pruning and quantization to address privacy, label scarcity, and resource limitations. Under open-set domain adaptation, Wu et al. [28] transferred intrusion knowledge from a labeled source domain to an unlabeled target IoT domain while identifying attack categories present only in the target domain.

These few-shot, online, federated, and domain-adaptation approaches reduce dependence on comprehensive labeled attack collections but rely on target-domain samples, labels, feedback, or post-deployment updates. They therefore support adaptation as information about an emerging family becomes available. By contrast, methods that do not rely on labeled attack samples can detect unusual behavior by modeling normal or predominant traffic patterns.

### 2.3. Detection Without Labeled Attack Information

Methods without labeled attack information may learn from a clean benign reference set or from an unlabeled distribution in which anomalies are assumed to be rare. These settings differ in their training assumptions, but both produce deviation evidence rather than probabilities tied to known attack classes. Isolation Forest identifies samples that can be separated through short random partitioning paths, whereas one-class representation methods such as Deep SVDD learn a compact region for normal data [14,15]. Reconstruction-based methods provide another major route: an autoencoder is trained to reproduce benign traffic, and large reconstruction errors are treated as anomaly evidence. N-BaIoT and Kitsune [7,12] demonstrated this strategy with device-level and ensemble autoencoders, respectively. Quantized autoencoders [29] further reduce memory and computation for resource-constrained IoT deployment.

Clustering-based approaches make the internal structure of benign traffic more explicit. IoT-KEEPER [20] combined fuzzy clustering and interpolation to detect malicious activity at an edge gateway, while Sivanathan et al. [21] model device traffic through clusters to identify behavioral change. These studies are relevant because legitimate IoT behavior need not form a single homogeneous distribution. Device roles, services, operating states, and capture scenarios can produce distinct traffic patterns, and scenario-dependent feature fluctuations have also been observed in IoT traffic analysis [13,22]. A normality model that represents this structure too narrowly may flag legitimate but less frequent behavior, whereas an overly broad representation may absorb malicious deviations.

Since labeled attack examples are unnecessary for fitting, benign-only and unsupervised methods can in principle respond to families absent from training. Their anomaly scores nevertheless indicate deviation rather than maliciousness, so attacks close to learned benign regions may remain undetected. These methods therefore complement, rather than replace, the discrimination learned from known attacks. This complementarity motivates the dual-evidence decision framework presented in Section 3.

### 2.4. Research Status Summary and Our Work

Table 1 summarizes the three routes, their development assumptions, and their main capabilities and limitations. Methods trained with labeled known attacks preserve discriminative information for represented families, whereas methods without labeled attack information can identify deviations from learned benign behavior. Limited-supervision and adaptive methods can combine or extend these capabilities when target-domain samples, labels, feedback, or model updates become available. The comparison shows that these routes address different operating conditions rather than forming a single uniformly superior solution.

In contrast, MCDE is designed for a complex setting, where samples from the held-out family remain unavailable throughout model development and the aggregate benign FPR must be controlled at a predefined low operating point. In MCDE, the supervised branch preserves known-attack discrimination, while the multi-mode benign-deviation branch provides an independent route for traffic poorly represented by learned normal behavior. The empirical rank alignment then makes the two heterogeneous scores comparable, and the branch-wise FPR budget with held-out threshold calibration enables explicit false-alarm control. Together, these mechanisms make MCDE suitable for deployment scenarios that require both sensitivity to unseen attacks and a guaranteed low false-positive rate.

## 3. Proposed Method

**Figure 1:**
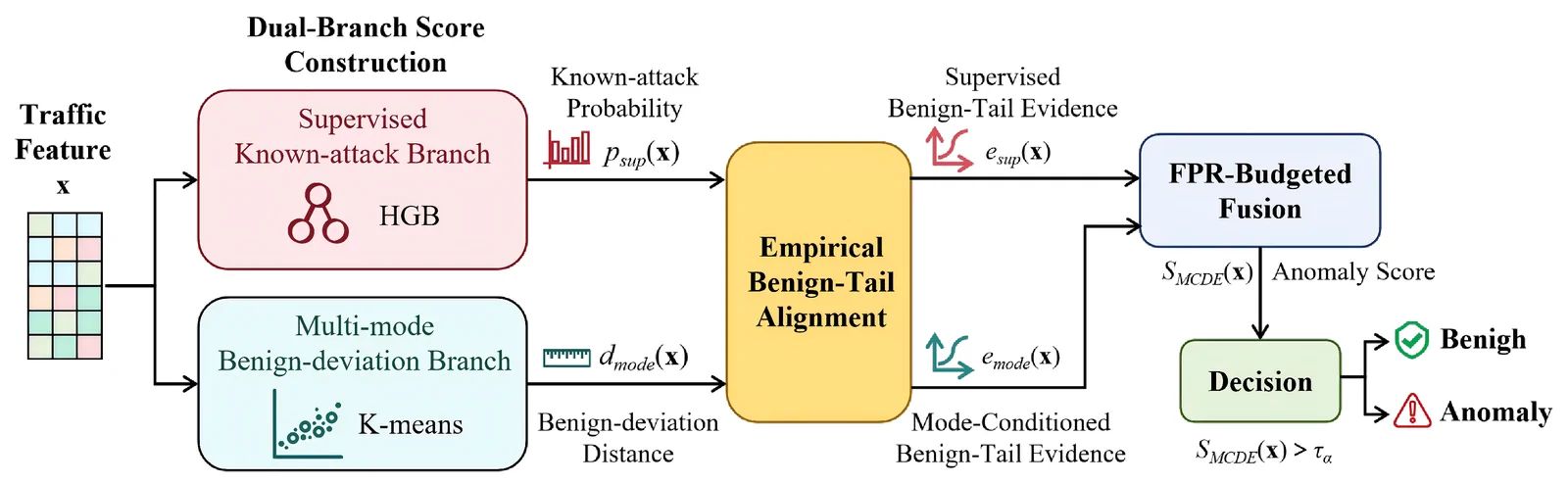
Overall detection workflow of MCDE. Colors are used to visually distinguish the functional components.

This section presents the Mode-Calibrated Dual-Evidence Detector (MCDE). Figure 1 gives the overall detection workflow. Given a traffic-derived feature vector, the fixed MCDE components produce two complementary raw scores: an estimated malicious-class probability from the supervised branch and a local distance indicating deviation from heterogeneous benign behavior. These scores are aligned through benign empirical references, fused under a nominal false-positive-rate (FPR) budget, and compared with a calibrated threshold to produce the final decision. The following subsections formulate the detection problem, explain how the two branch scores are constructed, derive the calibration and fusion mechanism, and present its information-theoretic interpretation and complete procedure.

### 3.1. Problem Formulation and Design Requirements

This subsection defines the unseen-family detection setting, states the low-FPR detection objective, and derives the corresponding design requirements for MCDE.

Let x∈X denote a traffic-derived feature vector and y∈{0,1} its binary label, where 0 denotes benign traffic and 1 denotes malicious traffic. The disjoint sets $A_{K}$ and $A_{U}$ contain the known and unseen attack families, respectively. The data are partitioned according to their roles in model development and evaluation. The benign set $B_{tr}$ and known-attack set $D_{K}^{tr}$ are used to fit the detection branches. Two disjoint benign sets are reserved for calibration: $B_{c1}$ constructs the empirical evidence references, whereas $B_{c2}$ determines the final decision threshold. Evaluation uses the held-out benign, known-attack, and unseen-attack sets $B_{te}$, $D_{K}^{te}$, and $D_{U}^{te}$, respectively.

Based on this partition, unseen status is defined at the attack-family level and depends on whether that family is absent from the entire development procedure.

**Definition 1** (Unseen attack family)**.** *An attack family belongs to $A_{U}$ if none of its samples is used for preprocessing fitting, branch fitting, empirical reference construction, or final-threshold calibration. Its samples occur only in $D_{U}^{te}$. Thus, “unseen” means that family-level attack information is absent from the entire development procedure, rather than only from classifier training. This setting is distinct from few-shot adaptation, online updating, and cross-dataset transfer*.

Under this protocol, MCDE must detect both known and unseen attacks while operating at a user-specified benign FPR target α. Let $S_{MCDE}\left(x\right)$ be a high-is-anomalous score and $\tau_{\alpha }$ its decision threshold. The desired operating condition is

(1) $$\underset{x∼P_{B}}{\mathrm{Pr}}\;\left[S_{MCDE}\left(x\right)>\tau_{\alpha }\right]\leq \alpha ,$$

where $P_{B}$ denotes the benign traffic distribution at deployment. Subject to this false-alarm constraint, the detector should retain high recall for attacks from both $A_{K}$ and $A_{U}$.

Meeting these two goals requires MCDE to satisfy three design requirements:

(i) Known-attack discrimination. The detector should preserve the discriminative information provided by labeled known attacks.
(ii) Unseen-deviation coverage. The detector should provide an additional detection route for traffic that is poorly represented by learned benign behavior, including attacks outside the supervised malicious region.
(iii) FPR-aware evidence fusion. The two routes must be combined without exceeding the requested benign false-alarm level. Because supervised probabilities and benign-deviation distances differ in semantics, scale, and tail behavior, they require a common evidence interpretation and an explicit allocation of the nominal FPR budget; direct averaging or maximum fusion provides neither.

The first two requirements motivate the dual-branch score construction in the next subsection. The third motivates the empirical tail alignment and FPR-budgeted fusion mechanism developed thereafter.

### 3.2. Dual-Branch Score Construction

This subsection describes the construction of the two raw scores produced by the branches in Figure 1. Let $\phi_{sup}$ and $\phi_{ben}$ denote the preprocessing maps used by the supervised and benign-deviation branches, respectively. We then clarify the complementary roles of the two scores and why they require alignment before fusion.

#### 3.2.1. Supervised Known-Attack Branch

The supervised branch models the boundary that can be learned directly from labeled benign and known malicious samples. An HGB classifier $h_{\theta }$ is fitted on $\left\{\left(\phi_{sup}\left(x\right),0\right):x\in B_{tr}\right\}$ and $\left\{\left(\phi_{sup}\left(x\right),1\right):x\in D_{K}^{tr}\right\}$ [30,31]. For an input x, it produces

(2) $$p_{sup}\left(x\right)=h_{\theta }\left(\phi_{sup}\left(x\right)\right)\in \left[0,1\right],$$

where a larger value indicates stronger classifier evidence that the sample is malicious. HGB is used because it is effective on tabular traffic features and inexpensive to fit and evaluate. This branch is expected to preserve high recall for $A_{K}$, but it may assign a low malicious probability to an unseen family outside the learned malicious region.

#### 3.2.2. Multi-Mode Benign-Deviation Branch

Benign IoT traffic is not assumed to form one homogeneous distribution. Devices, services, and operating states can produce several legitimate regions in feature space. MCDE applies mini-batch *K*-means to $\phi_{ben}\left(B_{tr}\right)$. With transformed dimension *q*, the fitted centroids ${\left\{c_{k}\right\}}_{k=1}^{K}$ approximately minimize

(3) $$\sum_{k=1}^{K}\sum_{x\in C_{k}}{∥\phi_{ben}\left(x\right)-c_{k}∥}_{2}^{2},$$

where $C_{k}$ is the set of benign vectors assigned to mode *k*. The modes summarize recurring benign communication patterns; they are not predefined device or attack classes. A diagonal scale vector $\sigma_{k}$ is estimated from each $C_{k}$. The global scale vector is first floored coordinate-wise by the larger of $10^{-3}$ and 5% of the median global standard deviation among coordinates exceeding $10^{-6}$; the floor is $10^{-3}$ if no coordinate exceeds $10^{-6}$. For feature *j*, its local scale is then lower-bounded by 5% of the corresponding floored global scale, and a mode containing fewer than 20 training vectors uses the global scale vector. These safeguards prevent constant coordinates or poorly populated modes from producing arbitrarily large standardized distances.

For a query vector, the distance to mode *k* is

(4) $$d_{k}\left(x\right)=\sqrt{\frac{1}{q}\sum_{j=1}^{q}{\left(\frac{\phi_{ben,j}\left(x\right)-c_{kj}}{\sigma_{kj}}\right)}^{2}}.$$

The assigned mode and raw benign-deviation score are

(5) $$m\left(x\right)=\arg\min_{1\leq k\leq K}d_{k}\left(x\right),\;d_{mode}\left(x\right)=d_{m(x)}\left(x\right).$$

Equation (4) is a root-mean-square diagonal Mahalanobis distance. The lower-bounding operation above ensures that every $\sigma_{kj}$ is positive. The distance measures deviation relative to the local variability of a benign mode instead of comparing all traffic with a single global center. Root-mean-square Euclidean distance is retained as an ablation to determine whether mode-specific scaling is necessary.

The two branches answer different questions. $p_{sup}\left(x\right)$ measures supervised evidence of maliciousness learned from labeled benign and known-attack traffic, whereas $d_{mode}\left(x\right)$ measures deviation from the nearest learned benign mode. Neither branch is sufficient in isolation, and their numerical values are not directly comparable. The next subsection aligns their meanings relative to benign traffic before the evidence is fused.

### 3.3. Empirical Benign-Tail Alignment

**Figure 2:**
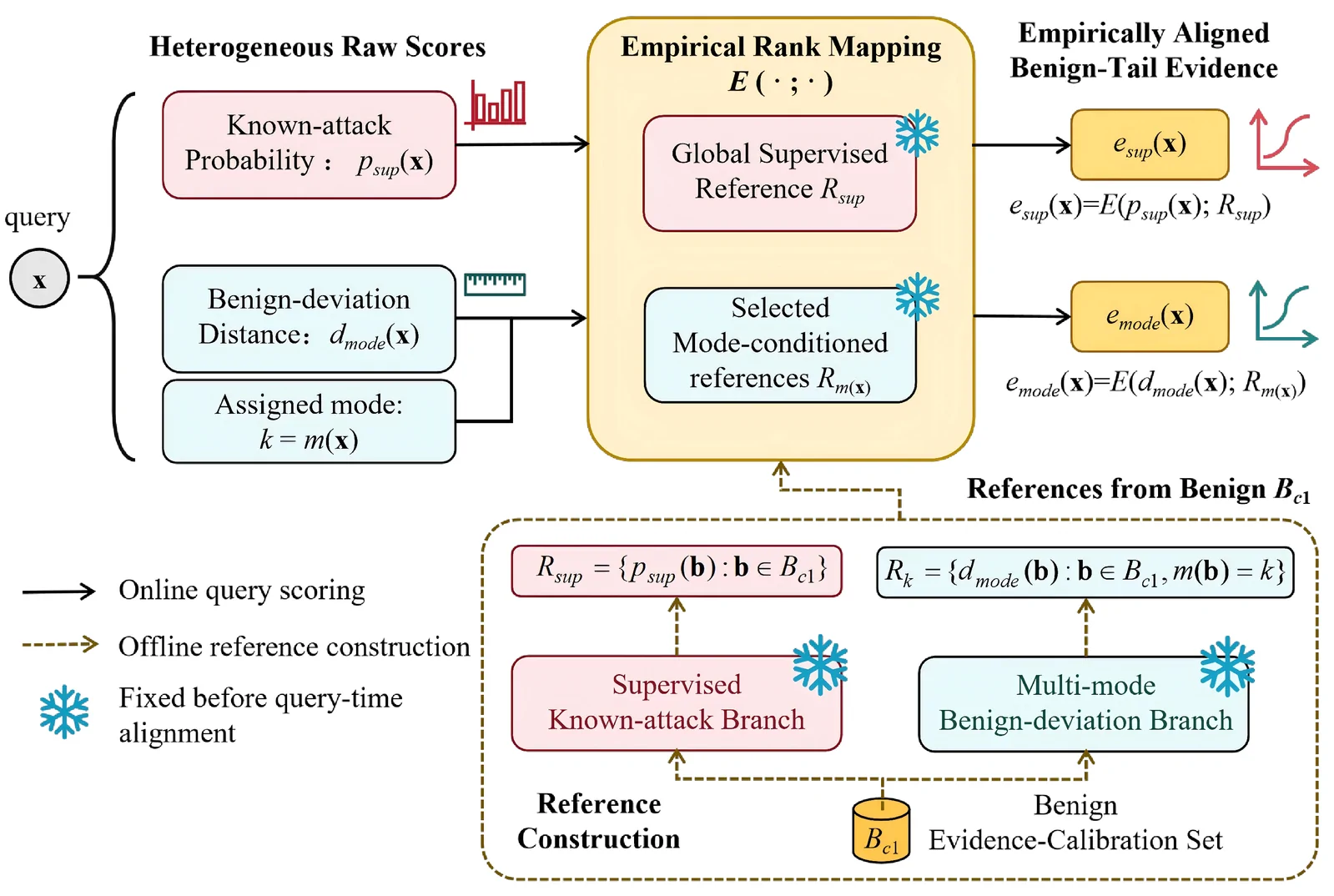
Empirical benign-tail alignment in MCDE. Dashed arrows show offline reference construction from $B_{c1}$, solid arrows show query-time mapping, and snowflakes denote fixed components. Each raw score is ranked against its corresponding benign reference to produce aligned evidence $e_{sup}\left(x\right)$ and $e_{mode}\left(x\right)$.

The two branches produce scores with different meanings and distributions. This subsection details the empirical benign-tail alignment module in Figure 1, which converts both scores into comparable evidence of extremeness relative to benign traffic. Figure 2 shows its offline reference construction and query-time rank mapping.

#### 3.3.1. Offline Reference Construction

The dashed path in Figure 2 constructs the benign references after both branches have been fitted. With the branch parameters fixed, every $b\in B_{c1}$ is scored in the same manner as a query. The supervised probabilities form one global reference, whereas the benign-deviation distances are partitioned by assigned mode:

(6) $$\begin{array}{cc} R_{sup} & =\{p_{sup}\left(b\right):b\in B_{c1}\},\\ R_{k} & =\{d_{mode}\left(b\right):b\in B_{c1},\;m\left(b\right)=k\},\;k=1,\ldots ,K. \end{array}$$

Here, $R_{sup}$ describes the benign-score distribution of the supervised branch, and $R_{k}$ describes the benign-distance distribution within mode *k*. A query assigned to a mode with insufficient calibration samples uses the pooled benign-distance reference over $B_{c1}$, avoiding an unstable local empirical distribution. The references are then stored for query-time mapping. The role of $B_{c1}$ is limited to reference construction; it does not refit either branch.

#### 3.3.2. Query-Time Empirical Rank Mapping

The solid path in Figure 2 maps a query after its two raw scores have been obtained. The known-attack probability $p_{sup}\left(x\right)$ is compared with the global supervised reference $R_{sup}$. The benign-deviation distance $d_{mode}\left(x\right)$ is compared with the mode-conditioned reference $R_{m(x)}$ selected by its assigned mode. For any high-is-anomalous score z(x) and corresponding benign reference $R={\left\{r_{i}\right\}}_{i=1}^{n}$, the empirical rank mapping is

(7) $$E\left(z\left(x\right);R\right)=\frac{1}{n+1}\sum_{i=1}^{n}I\left[r_{i}<z\left(x\right)\right].$$

This mapping measures the proportion of reference scores below the query score. The denominator n+1 prevents finite-sample evidence from reaching one, and the strict comparison handles ties conservatively. Applying Equation (7) to the two score–reference pairs gives

(8) $$\begin{array}{cc} e_{sup}\left(x\right) & =E\;\left(p_{sup}\left(x\right);R_{sup}\right),\\ e_{mode}\left(x\right) & =E\;\left(d_{mode}\left(x\right);R_{m(x)}\right). \end{array}$$

A value near one indicates that the query exceeds almost all benign scores in the corresponding reference. Thus, $e_{sup}\left(x\right)$ and $e_{mode}\left(x\right)$ preserve the complementary meanings of the two branches while sharing the same empirical benign-tail scale.

For subsequent FPR allocation, MCDE uses the complementary empirical upper-tail probability

(9) $$u\left(z\left(x\right);R\right)=1-E\left(z\left(x\right);R\right)=\frac{1+\sum_{i=1}^{n}I\left[r_{i}\geq z\left(x\right)\right]}{n+1},$$

This construction adapts the finite-sample rank-calibration principle used in inductive conformal anomaly detection [32,33]. MCDE applies it separately to the supervised score and the mode-conditioned benign-deviation score. It gives $u_{sup}\left(x\right)=1-e_{sup}\left(x\right)$ and $u_{mode}\left(x\right)=1-e_{mode}\left(x\right)$. The added unit and non-strict upper-tail count provide conservative treatment of finite samples and ties. If the scoring function is fixed independently of R and a future benign score is exchangeable with the reference scores, then ${\mathrm{Pr}}_{B}\left\{u\leq t\right\}\leq t$ for t∈[0,1]. For a supported mode, this property is conditional on the assigned mode. The pooled fallback improves rank resolution for sparsely calibrated modes but does not by itself inherit mode-conditional validity; its tail probabilities satisfy the subsequent control condition only when they remain super-uniform for benign traffic. These aligned tail probabilities provide the inputs to the FPR-budgeted fusion in the next subsection.

### 3.4. FPR-Budgeted Fusion and Calibrated Decision

This subsection presents the FPR-budgeted fusion and calibrated decision mechanism of MCDE in two parts. Section 3.4.1 allocates the target FPR α between the two evidence branches, constructs the budget-normalized fusion score, and proves that their combined nominal benign-alarm probability is bounded by α under valid benign-tail evidence, without requiring branch independence. Section 3.4.2 calibrates the final decision threshold on the disjoint held-out benign set $B_{c2}$ and establishes the resulting empirical FPR bound on that set. The two analyses clarify the respective roles and validity conditions of branch-wise budget allocation and final operating-point calibration.

#### 3.4.1. Branch-Wise FPR Budgeting and Theoretical Control

Let $u_{sup}\left(x\right)=1-e_{sup}\left(x\right)$ and $u_{mode}\left(x\right)=1-e_{mode}\left(x\right)$ denote the branch-specific tail probabilities defined by Equation (9). MCDE assigns wα of the nominal FPR budget to the supervised branch and (1−w)α to the mode branch, where 0<w<1. A branch can therefore trigger an alarm only when its tail probability falls below its allocated share. The following proposition gives the resulting joint bound.

**Proposition 1** (Nominal false-positive budget control)**.** *Suppose that, for benign traffic, $u_{sup}$ and $u_{mode}$ are super-uniform, so that ${\mathrm{Pr}}_{B}\left\{u_{j}<t\right\}\leq t$ for j∈{sup,mode} and all t∈[0,1]. For 0<w<1, define the nominal alarm event*

(10) $$A_{\alpha }=\left\{u_{sup}<w\alpha \right\}\cup \left\{u_{mode}<\left(1-w\right)\alpha \right\}.$$

*Then ${\mathrm{Pr}}_{B}\left(A_{\alpha }\right)\leq \alpha$, without requiring independence between the two branches.*

**Proof.**  By the union bound and branch-wise super-uniformity,

(11) $$\begin{array}{cc} \underset{B}{\mathrm{Pr}}\left(A_{\alpha }\right) & \leq \underset{B}{\mathrm{Pr}}\left\{u_{sup}<w\alpha \right\}+\underset{B}{\mathrm{Pr}}\left\{u_{mode}<\left(1-w\right)\alpha \right\}\\ \; & \leq w\alpha +(1-w)\alpha =\alpha . \end{array}$$

   □

Under its branch-wise super-uniformity condition, Proposition 1 formalizes why the allocation controls the combined nominal FPR. If both branches were allowed the full budget α, their union could have benign alarm probability as high as 2α under the same bound. Dividing the budget into wα and (1−w)α instead limits the sum to α. Thus, *w* is a benign-risk allocation rather than an unconstrained averaging weight. This result concerns the branch-triggered decision event itself and does not depend on subsequently fitting a threshold. It motivates the normalized tail statistic

(12) $$T_{MCDE}\left(x\right)=\min\left(\frac{u_{sup}\left(x\right)}{w},\frac{u_{mode}\left(x\right)}{1-w}\right)$$

and the equivalent high-is-anomalous fusion score

(13) $$S_{MCDE}\left(x\right)=-T_{MCDE}\left(x\right)=-\min\left(\frac{1-e_{sup}\left(x\right)}{w},\frac{1-e_{mode}\left(x\right)}{1-w}\right).$$

The primary configuration uses w=0.9, assigning most of the alert budget to the more discriminative supervised branch while retaining a stricter residual route for benign-mode deviation. This high supervised share was selected after preliminary low-diversity stress analysis showed that lower shares could severely suppress detection of a sparsely represented unseen family. At w=0.5, Equation (13) is a monotonic transformation of $\max(e_{sup},e_{mode})$; the latter is retained as the equal-priority dual-evidence ablation.

The fusion rule also admits a benign-tail surprisal interpretation. Define the empirical self-information, or benign-tail surprisal, of branch *j* as $I_{j}\left(x\right)=-\log u_{j}\left(x\right)$ [34]. Because the empirical tail probabilities are strictly positive,

(14) $$-\log T_{MCDE}\left(x\right)=\max\left\{I_{sup}\left(x\right)+\log w,I_{mode}\left(x\right)+\log\left(1-w\right)\right\}.$$

Under the nominal rule, a branch assigned a smaller FPR share must provide correspondingly greater benign-tail surprisal before it can trigger an alarm. Equation (14) is only a monotonic interpretation of the fusion statistic; it is not an additional entropy estimator, detection stage, or false-positive guarantee.

#### 3.4.2. Budget-Normalized Fusion and Final Calibration

Equation (13) retains evidence from either branch after normalizing its tail probability by the corresponding budget share. In particular, $T_{MCDE}\left(x\right)<\alpha$ is equivalent to the nominal alarm event in Proposition 1. The high-is-anomalous score $S_{MCDE}$ therefore increases when at least one branch provides evidence strong enough relative to its allocated benign-risk budget.

The nominal result relies on valid branch-tail probabilities. With finite references, mode-specific fallback, and sampling variation, directly applying the theoretical threshold −α may not attain the requested FPR in a realized dataset. MCDE therefore uses the second disjoint benign set $B_{c2}$ to calibrate the deployed threshold,

(15) $$\tau_{\alpha }=Q_{1-\alpha }^{\mathrm{higher}}\left(\{S_{MCDE}\left(b\right):b\in B_{c2}\}\right),$$

where $Q^{\mathrm{higher}}$ selects an observed upper quantile. The final output is

(16) $$\hat{y}\left(x\right)=\left\{\begin{array}{cc} 1, & S_{MCDE}\left(x\right)>\tau_{\alpha },\\ 0, & S_{MCDE}\left(x\right)\leq \tau_{\alpha }. \end{array}\right.$$

**Proposition 2** (Empirical final-calibration control)**.** *Let $\tau_{\alpha }$ be the higher empirical (1−α)-quantile in Equation *(15)*, and use the strict decision rule in Equation *(16)*. Then the empirical FPR on $B_{c2}$ satisfies*

(17) $${\hat{\mathrm{FPR}}}_{c2}=\frac{1}{|B_{c2}|}\sum_{b\in B_{c2}}I\left[S_{MCDE}\left(b\right)>\tau_{\alpha }\right]\leq \alpha .$$

**Proof.**  The higher empirical quantile selects an observed score no lower than the empirical (1−α)-quantile. Consequently, no more than an α fraction of the calibration scores can be strictly greater than $\tau_{\alpha }$; ties at the threshold are classified as benign.    □

The final-threshold set $B_{c2}$ is disjoint from $B_{c1}$, which was used to construct the empirical evidence mappings in the preceding subsection. The final operating point is therefore not selected on the same benign samples used for evidence construction. Proposition 1 establishes a conditional population-level bound for the nominal branch event under valid benign-tail probabilities, whereas Proposition 2 establishes an empirical bound on $B_{c2}$. Extending these bounds to future traffic requires compatibility between the deployment and calibration benign-score distributions; they do not constitute unconditional guarantees under network or temporal distribution shift.

Section 3.2, Section 3.3 and Section 3.4 now define the complete MCDE decision process. For each input x, the fitted detector returns the budget-normalized anomaly score $S_{MCDE}\left(x\right)$ and the final benign-or-malicious decision $\hat{y}\left(x\right)$ at the requested operating point α. The next subsection summarizes the complete offline construction and query-time detection procedure without introducing an additional detection stage.

### 3.5. Algorithmic Summary

Algorithm 1 complements the overview in Figure 1 by consolidating branch fitting, reference construction, threshold calibration, and query-time detection in their execution order. Attack labels are used only to fit the supervised branch; benign-mode fitting and both calibration stages use benign data. All fitted components and calibration references remain fixed during detection.
**Algorithm 1:** MCDE Procedure      **Require:** $B_{tr}$, $D_{K}^{tr}$, $B_{c1}$, $B_{c2}$, *K*, α, and *w*.  1  Fit $\phi_{sup}$ on $B_{tr}\cup D_{K}^{tr}$ and $\phi_{ben}$ on $B_{tr}$.  2  Fit the HGB branch $h_{\theta }$ using transformed benign and known-attack samples with      binary labels.  3  Fit *K* benign modes and their diagonal scale vectors using $\phi_{ben}\left(B_{tr}\right)$.  4  Score $B_{c1}$ with both branches and construct $R_{sup}$.  5  **for** each mode k=1,…,K **do**  6     Construct $R_{k}$ from its assigned benign scores; use the pooled reference if the mode    is sparse.  7  **end for**  8  **for** each $b\in B_{c2}$ **do**  9     Map both branch scores to $e_{sup}\left(b\right)$ and $e_{mode}\left(b\right)$ using Equation (7).10     Compute $S_{MCDE}\left(b\right)$ using Equation (13).11  **end for**12  Set $\tau_{\alpha }$ to the higher empirical (1−α)-quantile of $\left\{S_{MCDE}\left(b\right):b\in B_{c2}\right\}$.13  **for** each query x **do**14     Compute $p_{sup}\left(x\right)$, assign m(x), and compute $d_{mode}\left(x\right)$.15     Map $p_{sup}\left(x\right)$ using $R_{sup}$.16     **if** mode m(x) is supported **then**17        Map $d_{mode}\left(x\right)$ using $R_{m(x)}$.18     **else** map $d_{mode}\left(x\right)$ using the pooled reference.19     **end if**20     Compute $S_{MCDE}\left(x\right)$ and set $\hat{y}\left(x\right)=I\left[S_{MCDE}\left(x\right)>\tau_{\alpha }\right]$.21     **return** $S_{MCDE}\left(x\right)$ and $\hat{y}\left(x\right)$.22  **end for**      **Ensure:** Fitted MCDE components, threshold $\tau_{\alpha }$, and $\left(S_{MCDE}\left(x\right),\hat{y}\left(x\right)\right)$ for each      query x.

### 3.6. Computational Complexity

This subsection analyzes the computational and storage costs introduced by MCDE and provides the complexity basis for the CPU-only efficiency evaluation in Section 4.8.

Let $n_{tr}=\left|B_{tr}\right|$, $n_{K}=\left|D_{K}^{tr}\right|$, $n_{c1}=\left|B_{c1}\right|$, and $n_{c2}=\left|B_{c2}\right|$. Let $q_{sup}$ and $q_{ben}$ be the transformed dimensions of the two branches, and let *K* be the number of benign modes. For fixed preprocessing maps, transformation costs are linear in the number of samples and transformed dimensions. HGB fitting on $n_{tr}+n_{K}$ samples is implementation- and tree-structure-dependent. The additional benign-branch cost is dominated by MiniBatch *K*-means, with approximate update cost $O(UbKq_{ben})$, followed by full mode assignment at $O(n_{tr}Kq_{ben})$ and diagonal scale estimation at $O(n_{tr}q_{ben})$.

Constructing the empirical references costs $O(n_{c1}Kq_{ben})$ for mode scoring and $O(n_{c1}\log n_{c1})$ for dominant sorting, in addition to HGB scoring. Threshold calibration adds $O(n_{c2}Kq_{ben})$ distance computation, fusion, and empirical quantile selection. For one query, mode assignment costs $O(Kq_{ben})$; HGB inference depends on the fitted classifier, and fusion is constant-time. If references are sorted once, lookup against a reference of size $n_{ref}$ costs $O(\log n_{ref})$. The current implementation sorts each supplied reference before searchsorted, and this cost is included in the measured throughput. and this cost is included in the measured throughput. MCDE stores $O(Kq_{ben}+n_{c1})$ mode parameters and benign reference scores, in addition to the HGB model and preprocessing objects. Section 4.8 measures these costs under a controlled CPU-only protocol, including model fitting, inference throughput, process memory, and deployment-bundle size.

## 4. Experiments

This section evaluates MCDE on two public IoT traffic datasets under attack-family hold-out protocols. After introducing the datasets and common experimental setup, we report the main unseen-family results, complementary stress tests, ablations, sensitivity analyses, and computational efficiency.

### 4.1. Datasets

We evaluate MCDE on N-BaIoT, IoT-23, and CICIoT2023. The first two datasets represent complementary forms of IoT traffic data: device-oriented statistical features (N-BaIoT) and scenario-oriented connection records (IoT-23). Their different collection procedures, attack taxonomies, and native feature representations allow us to evaluate the decision framework without forcing the datasets into an artificial shared feature space. CICIoT2023 is used as an additional cross-dataset validation in Section 4.5. Table 2 summarizes the three datasets.

**N-BaIoT [7]:** N-BaIoT was collected from nine commercial IoT devices that generated benign traffic and were subsequently infected with the Gafgyt (BASHLITE) and Mirai botnets. The public dataset contains 7,062,606 traffic records. Gafgyt is represented by combo, junk, scan, TCP, and UDP attacks, whereas Mirai is represented by ACK, scan, SYN, UDP, and UDP-plain attacks. Each record contains 115 numerical traffic statistics extracted over five exponentially decayed time windows. These features describe traffic associated with source hosts, host pairs, host–port pairs, and inter-packet timing. We retain all 115 native numerical features.

**IoT-23 [35]:** IoT-23 contains 23 scenarios, including 20 malware-infection scenarios and three benign scenarios. The dataset provides packet captures and labeled Zeek connection logs. We use the labeled conn.log.labeled records rather than raw packets. The controlled experiments retain DDoS, horizontal port scanning, C&C, and Okiru traffic. DDoS, horizontal port scanning, and C&C form the main family-hold-out set, whereas Okiru is reserved for the rare-family stress test in Section 4.5. Each IoT-23 input vector contains 16 connection-level fields: 10 numerical fields and six categorical fields.

**CICIoT2023 [36]:** CICIoT2023 is a recent IoT traffic dataset collected from a testbed of 105 IoT devices, covering 33 attack types organized into seven categories. The dataset provides 46 flow-level features extracted from captured network traffic. For the cross-dataset validation experiment in Section 4.5, we retain the 39 available flow-level features and focus on three attack categories: DDoS, DoS, and Mirai.

### 4.2. Experimental Setup

The experimental setup is designed to isolate attack-family novelty while reserving disjoint held-out benign splits for evidence construction, threshold calibration, and final evaluation. The following subsections describe data preparation and partitioning, the common operating configuration, and the implementation environment.

#### 4.2.1. Data Preprocessing and Partitioning

**Attack-family isolation.** The held-out attack family is removed before any feature transformation, detector fitting, calibration, or threshold selection. On N-BaIoT, Gafgyt and Mirai are withheld in turn; on IoT-23, DDoS, port scanning, and C&C are withheld in turn, while Okiru is reserved as a rare-family stress case. Generic and composite labels are excluded from the main hold-out protocol. This ensures that the target family is absent throughout development and that evaluation measures genuine unseen-family detection.

**Leakage and identifier control.** Duplicate feature vectors and multi-label samples are removed before partitioning. N-BaIoT uses all 115 numerical features. IoT-23 uses the 10 numerical and six categorical connection fields. IP addresses, timestamps, labels, and capture identifiers are excluded from model inputs. Capture or device identifiers are retained only for grouped splitting and group-wise FPR analysis. These steps prevent data leakage from duplicate records, conflicting labels, or identifier shortcuts.

**Dataset-specific feature transformation.** Numerical values are median-imputed, transformed using signed log(1+|x|), and robustly scaled according to the 5th–95th percentile range. IoT-23 categorical values are mode-imputed and one-hot encoded with a categorical block weight of 0.5. The supervised preprocessor is fitted using benign and known-attack training data, whereas the benign-deviation preprocessor is fitted using benign training data only.

**Partition and sampling control.** Benign traffic is split into 60% training, 10% branch calibration, 10% threshold calibration, and 20% testing for each N-BaIoT device. IoT-23 benign captures use 45%, 30%, 12.5%, and 12.5%, respectively. Known attack pools are divided equally into training and testing; all held-out family samples are reserved for unseen testing. Attack pools are capped by type or family to prevent large groups from dominating fitting or evaluation. These controls keep the comparison feasible, reduce class-imbalance effects, and preserve the known/unseen distinction.

The resulting primary-run caps are summarized in Table 3.

#### 4.2.2. Operating Configuration

The primary operating point is a requested benign FPR of α=1%, with additional thresholds at α∈{0.5%,2%,5%}. The main MCDE configuration uses K=15 benign modes with diagonal Mahalanobis distance and a supervised budget share of w=0.9. The value of *K* is chosen based on the expected benign heterogeneity indicated by device categories and operating states, rather than tuned per held-out family. The three main IoT-23 family hold-outs and the two N-BaIoT family hold-outs are repeated with seeds 42, 7, and 2026.

The sparse-mode minimum is fixed at $\left\lceil 5/\alpha_{min}\right\rceil$, where $\alpha_{min}$ is the smallest target FPR evaluated, giving 1000 samples for the main and sensitivity runs and 500 for the 1%-only benign-device transfer study; smaller modes fall back to the global benign reference.

Neural baselines use batch size 2048, gradient clipping at 5, and 10 pretraining epochs for autoencoders. Hyperparameters (learning rate, epoch count, and clipping bound) are selected per N-BaIoT hold-out protocol using known-attack validation data and a benign subset held out from training; the held-out family and final calibration/test sets are excluded from this selection. For the Gafgyt hold-out, settings are $\left(3\times 10^{-4},50,50\right)$ for MLP and $\left(10^{-3},50,20\right)$ for AE, Deep SVDD, and Deep SAD. For the Mirai hold-out, settings are $\left(3\times 10^{-4},50,50\right)$ for MLP, $\left(10^{-3},50,20\right)$ for AE and Deep SAD, and $\left(10^{-4},50,20\right)$ for Deep SVDD.

#### 4.2.3. Baselines

We compare MCDE with seven external baselines. HGB and XGBoost represent gradient-boosted tabular classifiers motivated by their use in IoT malicious-access detection and smart-factory anomaly detection, respectively [24,37]. MLP represents supervised neural classification and has been evaluated in ensemble intrusion detection for IoT and fog-computing environments [23]. Isolation Forest provides an unsupervised isolation baseline for IoT traffic, whereas AE represents reconstruction-based detection [7,12,29]. Deep SVDD and Deep SAD provide one-class and semi-supervised deep anomaly baselines, respectively [26,38].

Every baseline uses the same dataset-specific feature fields, data partitions, final-threshold calibration split, and requested operating point as MCDE. Therefore, all numerical results reported in this paper are obtained from our own controlled experiments rather than copied from the cited studies. We also evaluate global distance, mode-calibrated distance, and alternative MCDE fusion rules as internal ablations.

#### 4.2.4. Implementation Environment

Experiments were conducted on an Ubuntu Linux 6.8 server with an AMD EPYC 7343 processor, 251.7 GiB RAM, and three NVIDIA GeForce RTX 4090 GPUs with 24,564 MiB memory each. The software environment comprised Python 3.10.20, scikit-learn 1.7.2, XGBoost 3.2.0, and PyTorch 2.6.0 with CUDA 12.4. The neural effectiveness experiments used one CUDA device. For the efficiency benchmark, all GPUs were masked and every method was executed on the same CPU with eight threads. The OMP_NUM_THREADS, MKL_NUM_THREADS, and OPENBLAS_NUM_THREADS variables were fixed to 8.

### 4.3. Evaluation Metrics and Reporting Protocol

Let B, $D_{K}$, and $D_{U}$ denote the benign, known-attack, and unseen-attack test sets, with sizes $N_{B}$, $N_{K}$, and $N_{U}$. For each detector, S(x) is its anomaly score, $\tau_{\alpha }$ its threshold at target FPR α, and I[·] the indicator function. Group-wise metrics use *g* to index a benign device or capture group, with $n_{g}$ benign test samples. Results are reported at α=1% unless otherwise stated; each table and figure specifies its aggregation unit and number of runs. Table 4 summarizes the metrics and their roles.

Normal FPR, $R_{K}$, $R_{U}$, and $H_{KU}$ are the primary operating metrics. Unseen AUROC and AUPRC provide complementary ranking diagnostics for the benign–unseen comparison. AUPRC is interpreted under the specified test composition because it depends on class proportions. Neither metric guarantees high recall at a fixed low-FPR threshold, as illustrated by the Okiru stress test. Worst supported-group FPR and group-FPR standard deviation reveal concentrated or uneven false alarms that aggregate FPR can hide. The main text reports aggregate performance, group-wise FPR, and per-label recall.

### 4.4. Main Unseen-Family Detection Experiment

This experiment tests whether a detector can identify an attack family that is completely absent during model development. For N-BaIoT, Gafgyt and Mirai are held out in separate runs. For IoT-23, DDoS, PortScan, and C&C are held out separately, while Okiru is excluded from this experiment because its limited support requires a dedicated rare-family stress test in Section 4.5. For each run, the held-out family is used only for final testing and is excluded from preprocessing, fitting, evidence construction, and threshold calibration. All methods use identical family-level partitions and three random seeds (42, 7, and 2026). Results are macro means over six family–seed runs for N-BaIoT and nine for IoT-23.

**Figure 3:**
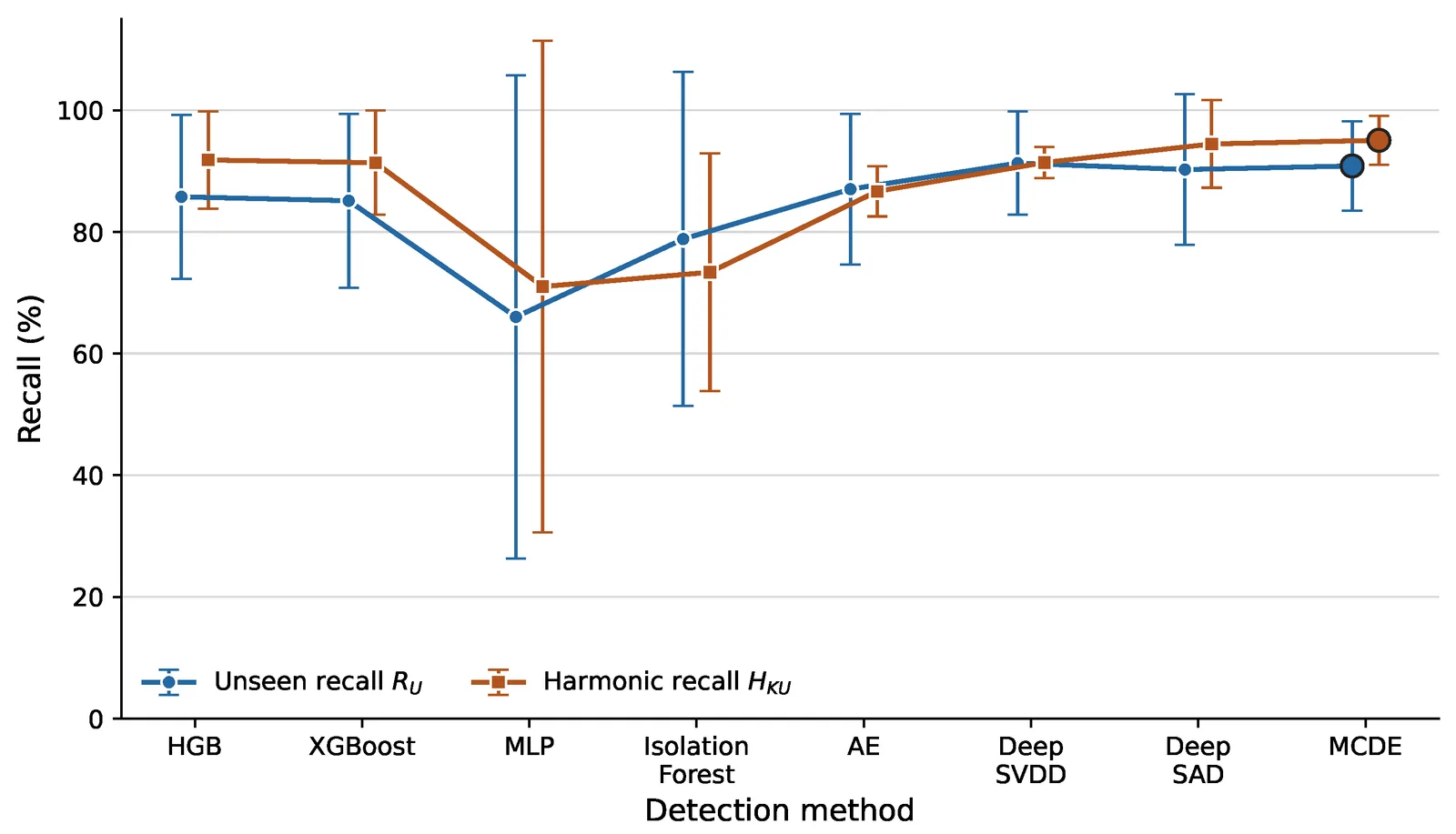
IoT-23 family-hold-out results at a 1% target benign FPR. The horizontal axis lists the compared detection methods, and the two curves show macro-mean unseen recall and harmonic known–unseen recall. Markers show macro means and error bars show one standard deviation over nine family–seed runs.

**Results on IoT-23.** Table 5 reports the aggregate results, and Figure 3 visualizes the macro-mean unseen recall and harmonic known–unseen recall. Three findings can be found. (i) HGB and XGBoost retain near-perfect known recall but obtain only 85.77% and 85.13% unseen recall, showing that strong known-attack discrimination does not ensure unseen-family detection. (ii) MCDE increases unseen recall to 90.85% while retaining 100% known recall, achieving an $H_{KU}$ of 95.07% at a normal FPR of 0.96%. Paired differences favor MCDE, but the nine family–seed pairs are not treated as sufficient evidence of statistical superiority. (iii) Deep SAD is the strongest balanced baseline, but MCDE achieves higher $R_{U}$ and $H_{KU}$ with lower and less variable worst supported-capture FPR. The main comparison supports MCDE’s balance of known- and unseen-attack detection.

**Results on N-BaIoT.** Table 6 shows near-ceiling performance for HGB, XGBoost, MLP, Deep SAD, and MCDE, whereas AE and Deep SVDD vary more across family–seed runs. MCDE obtains 99.87% unseen recall, 99.94% $H_{KU}$, and a normal FPR of 0.97%. These consistently high results reflect the strong separability of the 115 device-specific statistical features. In this setting, MCDE preserves the high detection performance achieved by the strongest baselines while maintaining explicit low-FPR calibration. Its worst-device FPR is lower than that of HGB but higher than that of Deep SAD, so MCDE should not be interpreted as a universal minimizer of group-wise false alarms.

Overall, the IoT-23 results provide the main evidence that MCDE improves the balance between known- and unseen-attack detection under a compact connection-level representation. The N-BaIoT results show that the same calibrated decision mechanism preserves near-ceiling performance when the native feature space already provides strong attack separability. Taken together, these results provide direct empirical support for the problem formulated in the Introduction: under the family-hold-out protocols, MCDE retains known-attack discrimination while providing complementary sensitivity to unseen attack families at a controlled low-FPR operating point.

### 4.5. Stability Under Complementary Evaluation Conditions

The main experiment evaluates complete family-hold-out under the standard in-domain benign calibration protocol. We further test MCDE under complementary conditions: attack-type hold-out, a low-diversity unseen family, benign-device calibration transfer, and cross-dataset validation on CICIoT2023.

**Fine-grained N-BaIoT attack-type hold-out.** This supplementary experiment holds out one attack type at a time from the five Gafgyt and five Mirai types, while the remaining attack types and the other family remain available for development. Each held-out type is excluded from preprocessing, fitting, evidence construction, and threshold calibration, and is used only for unseen testing. All runs use seed 42.

**Figure 4:**
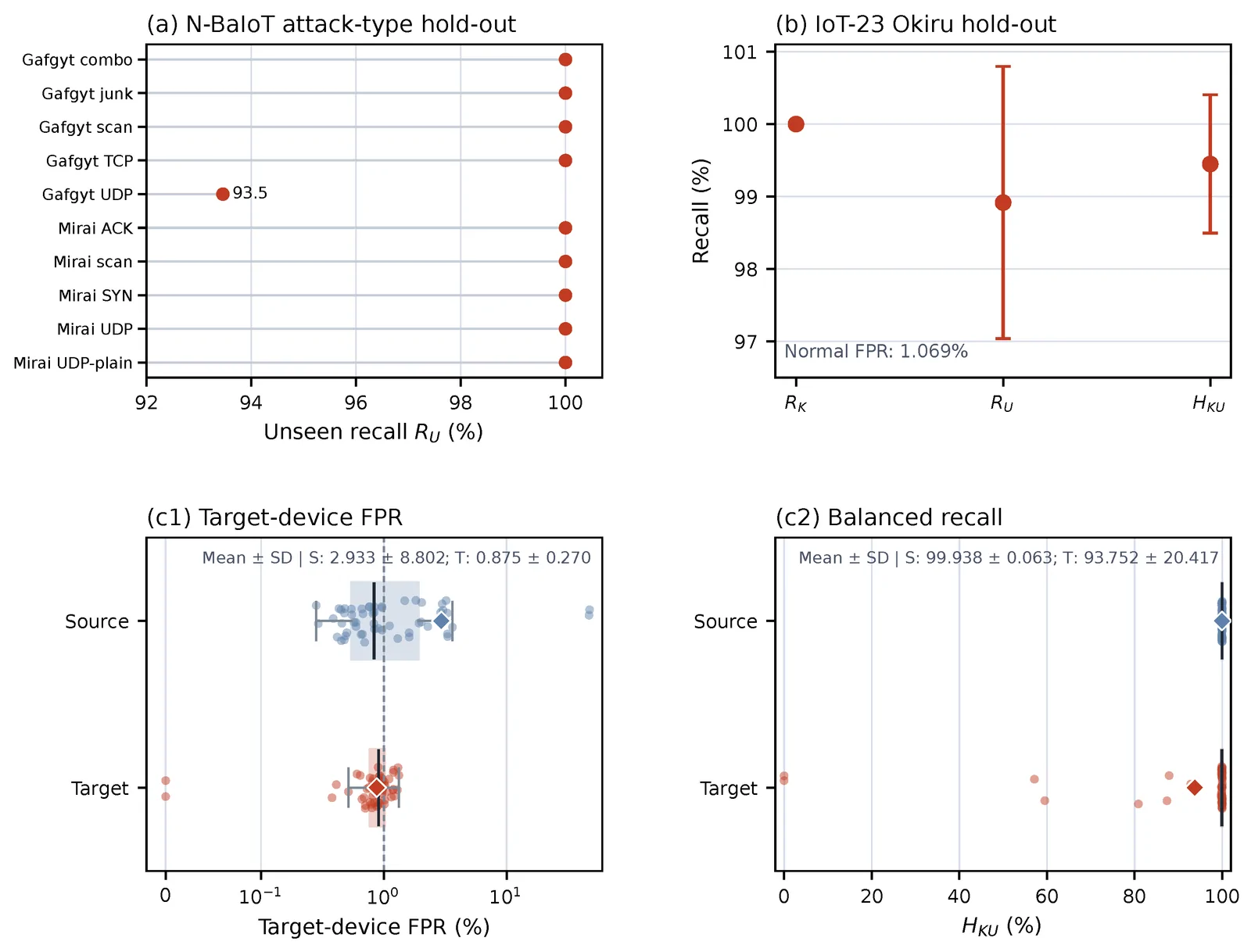
Stability of the final MCDE configuration under the first three complementary conditions. (**a**) Per-type unseen recall for the N-BaIoT attack-type hold-out at a 0.5% target FPR. (**b**) Known, unseen, and harmonic recall for the low-diversity IoT-23 Okiru hold-out at a 1% target FPR; points show means and error bars show one standard deviation over three seeds. (**c1**,**c2**) Distributions of target-device FPR and $H_{KU}$ over 54 paired N-BaIoT device–family–seed runs. Each point denotes one run, boxes show the interquartile range and median, and diamonds show the mean. S and T denote the source threshold and target-device recalibration, respectively. The dashed line in (**c1**) marks the 1% target FPR, and its horizontal axis uses a symmetric logarithmic scale.

At the 1% target FPR, MCDE achieves 100% unseen recall for every held-out attack type. Figure 4a therefore reports the more restrictive 0.5% operating point, at which the macro unseen recall is 99.35%. Nine attack types retain 100% recall, whereas Gafgyt UDP obtains 93.46%. This shows that MCDE generalizes from observed attack types to an unobserved type within a partially observed family, and that this protocol provides an easier test than complete family hold-out.

**Low-diversity IoT-23 unseen-family hold-out.** This condition tests whether MCDE remains operational when an unseen family occupies a narrow region of the selected feature space. Okiru is selected because, among 60,990,711 labeled records, a 450,000-record reservoir yields only 492 distinct vectors after deduplication in the 16-field representation. The three main IoT-23 families are used for development; all retained Okiru vectors are reserved for unseen testing. No Okiru vector is used for development.

Figure 4b shows that MCDE achieves 99.999% known-attack recall, 98.916% unseen-attack recall, and 99.449% $H_{KU}$, with a benign-test FPR of 1.069%. These results indicate that MCDE remains effective when the unseen family has limited feature-pattern diversity. This experiment is reported as a boundary-condition test and is excluded from the main IoT-23 family-hold-out average.

**N-BaIoT benign-device calibration transfer.** This experiment evaluates whether a threshold calibrated on source-device benign traffic remains valid for an excluded target device. Each of the nine N-BaIoT devices is treated as the target in turn. Its benign samples are excluded from mode fitting, evidence construction, and source-threshold calibration; the remaining eight devices provide source benign data. The target-device benign pool is split into a trusted 20% recalibration subset and a held-out 80% test subset. We compare the original source-calibrated threshold with a threshold recalibrated using only the trusted target subset. Branch models, evidence references, and attack scores remain unchanged. Nine target devices, two family hold-outs, and three seeds produce 54 paired runs per threshold condition.

Panels (c1) and (c2) of Figure 4 summarize the 54 paired runs. Panel (c1) shows that the source threshold yields a target-device FPR of 2.933±8.802%: the median is 0.832%, but 20 of 54 runs exceed 1%, 13 exceed 2%, and the maximum reaches 47.384%. Target-device recalibration reduces the FPR to 0.875±0.270%, with a median of 0.904% and a maximum of 1.324%. Thus, recalibration makes the FPR distribution substantially more stable. Panel (c2) shows the associated detection trade-off. With the source threshold, $H_{KU}$ is 99.938±0.063% and remains above 99% in every run. After target recalibration, the median $H_{KU}$ is 99.921%, the IQR is 99.833–100%, and 46 of 54 runs remain above 99%. However, eight runs fall below 99%, including four below 80%, reducing the mean to 93.752±20.417%. Thus, MCDE behaves stably in most target-device conditions but shows marked performance reductions in a small number of cases. Threshold-only recalibration improves local FPR control but does not guarantee preserved attack recall under every benign-device shift.

**Cross-dataset validation on CIC IoT 2023.** To examine whether the observed behavior extends beyond IoT-23 and N-BaIoT, we conduct a compact family-hold-out stress test on CIC IoT 2023 using its 39 extracted flow-level features. The evaluation focuses on three attack categories: DDoS, DoS, and Mirai. One of the three families is held out in turn, while the other two remain available during model development. MCDE is trained from scratch for held-out family with K=15, diagonal Mahalanobis distance, w=0.9, a 1% target FPR, and three random seeds. Table 7 reports the results.

Across the three held-out families, MCDE obtains an average known recall of 84.810% and an average unseen recall of 99.997%, with a normal FPR of 0.940% and an $H_{KU}$ of 91.720%. The near-perfect unseen recall confirms the effectiveness of MCDE on this additional dataset. The lower average known recall is mainly attributable to the DDoS hold-out case, suggesting that performance still depends on the held-out family and dataset characteristics. These results partially demonstrate the cross-dataset generalization capability of MCDE, but they do not imply that it possesses unconditional robustness.

Across four experiments, MCDE maintains high detection performance under diverse unseen conditions (varying unseen units, low-diversity families, and independent datasets). The transfer experiment reveals a complementary limitation: a source-calibrated threshold may cause concentrated false alarms on a target device. Recalibration reduces target FPR but cannot guarantee exactly 1% and may lower attack recall in shifted conditions. Thus, these results support an empirical, in-domain calibration claim, not an unconditional cross-device guarantee. Robust deployment may require updating references or benign modes beyond threshold adjustment.

### 4.6. Ablation Study on IoT-23

This experiment evaluates the contributions of benign-mode modeling, supervised evidence, and fusion strategy. Six variants are compared with MCDE: V1 uses global benign-distance without supervised evidence; V2 uses mode-calibrated distance without supervised evidence; V3 uses only the supervised HGB branch; V4 combines HGB with calibrated global-distance evidence; V5 and V6 combine HGB with mode-calibrated evidence using mean and maximum fusion, respectively. MCDE uses the same two evidence branches with FPR-budgeted fusion.

All configurations share identical splits, preprocessing, and threshold calibration. Mode-based variants use K=15 and diagonal Mahalanobis distance. Results are reported over nine main-family runs, three Okiru runs, and all twelve runs combined. IoT-23 is used because its non-saturated hold-outs provide sufficient separation among variants; the corresponding results on N-BaIoT are near-saturated.

Table 8 supports three conclusions. First, mode-specific modeling (V2) improves three-family $H_{KU}$ over global modeling (V1) from 89.05% to 92.94%, but distance-only variants perform poorly on Okiru, showing that benign-deviation evidence alone is insufficient. Second, the supervised branch performs reliably on Okiru, while combining it with benign-deviation evidence generally improves main-family results, confirming complementarity. Third, fusion strategy is critical: maximum fusion (V6) achieves the highest three-family $H_{KU}$ (95.67%) but collapses on Okiru (13.40%). MCDE balances both, achieving 95.07% on main families and 99.45% on Okiru, yielding the highest four-condition macro $H_{KU}$ (96.16%) with relatively low variation. This supports FPR-budgeted fusion as the preferred configuration among the evaluated variants.

### 4.7. Parameter and Operating-Point Sensitivity

This experiment characterizes MCDE’s sensitivity to the supervised FPR-budget share, benign-mode configuration, and target-FPR operating point.

#### 4.7.1. Parameter Sensitivity

We examine the supervised FPR-budget share, the number of benign modes *K*, and the distance metric. The share controls the relative benign-risk allowance of the two evidence branches, whereas *K* and the distance metric control the granularity and geometry of the benign-deviation branch.

**Supervised FPR-budget share.** The share *w* allocates the nominal FPR budget between the supervised and benign-deviation branches. We examine this parameter to understand the trade-off between known-attack discrimination and sensitivity to weakly represented unseen families, particularly under low-support conditions. At the 1% target FPR and K=15, we evaluate w∈{0.1,0.2,0.3,0.5,0.7,0.8,0.9,0.95,0.99} on the three principal IoT-23 hold-outs and on Okiru, a low-support unseen-family stress test derived from IoT-23. Table 9 summarizes the results. On the principal IoT-23 families, lower shares yield slightly higher $R_{U}$ and $H_{KU}$ (92.97% and 96.27% at w=0.1 versus 90.85% and 95.07% at w=0.9). On Okiru, however, unseen recall rises sharply from 4.95% at w=0.5 to 98.92% at w=0.9. On N-BaIoT, all shares remain near saturation ($H_{KU}$: 99.83–99.94%). No single share is optimal across all conditions; we retain w=0.9 as a practical compromise that preserves known-attack discrimination while substantially improving sensitivity to low-support unseen families.

**Number of benign modes.** In MCDE, *K* is the number of local traffic modes learned from benign feature vectors, rather than the number of devices or attack classes. A small *K* may merge distinct communication patterns, whereas an excessively large *K* may create sparsely populated modes and unstable distance estimates. Device categories and expected operating states provide prior guidance for choosing a common *K*, although they do not determine it exactly because one device may exhibit multiple modes and different devices may share similar traffic patterns. The sensitivity analysis below checks whether the results depend strongly on the preselected value K=15.

**Figure 5:**
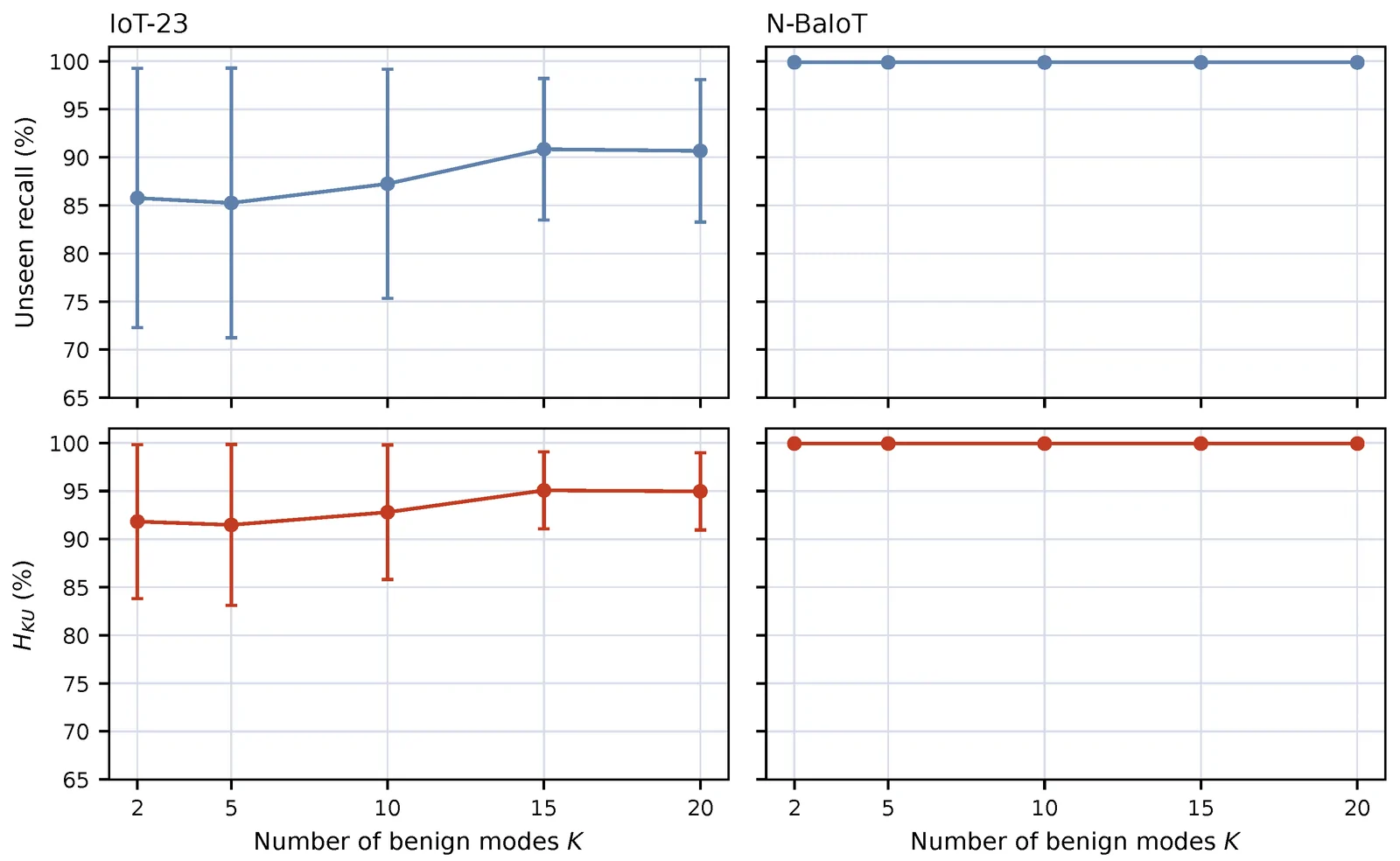
Sensitivity to the number of benign modes at the 1% target benign FPR.

We vary K∈{2,5,10,15,20} with diagonal Mahalanobis distance at the 1% target FPR, using the same *K* across all held-out families. Figure 5 shows that IoT-23 performance improves from K=5 to K=15. The nearly identical results at K=20 indicate a stable region rather than an isolated optimum. N-BaIoT varies by less than 0.02 percentage points across the tested range.

**Distance metric.** The distance metric determines how deviations along different feature dimensions contribute to the benign-mode score. We compare root-mean-square Euclidean and diagonal Mahalanobis distance at K=15 and the 1% target FPR using seed-42 macro results. Table 10 shows that diagonal Mahalanobis improves IoT-23 unseen recall from 88.07% to 90.86% and unseen AUROC from 95.82% to 99.63%, while both are near saturation on N-BaIoT. We retain K=15 and diagonal Mahalanobis distance: K=15 lies in the stable high-performing region and was used without family-specific tuning.

#### 4.7.2. Operating-Point Sensitivity

**Figure 6:**
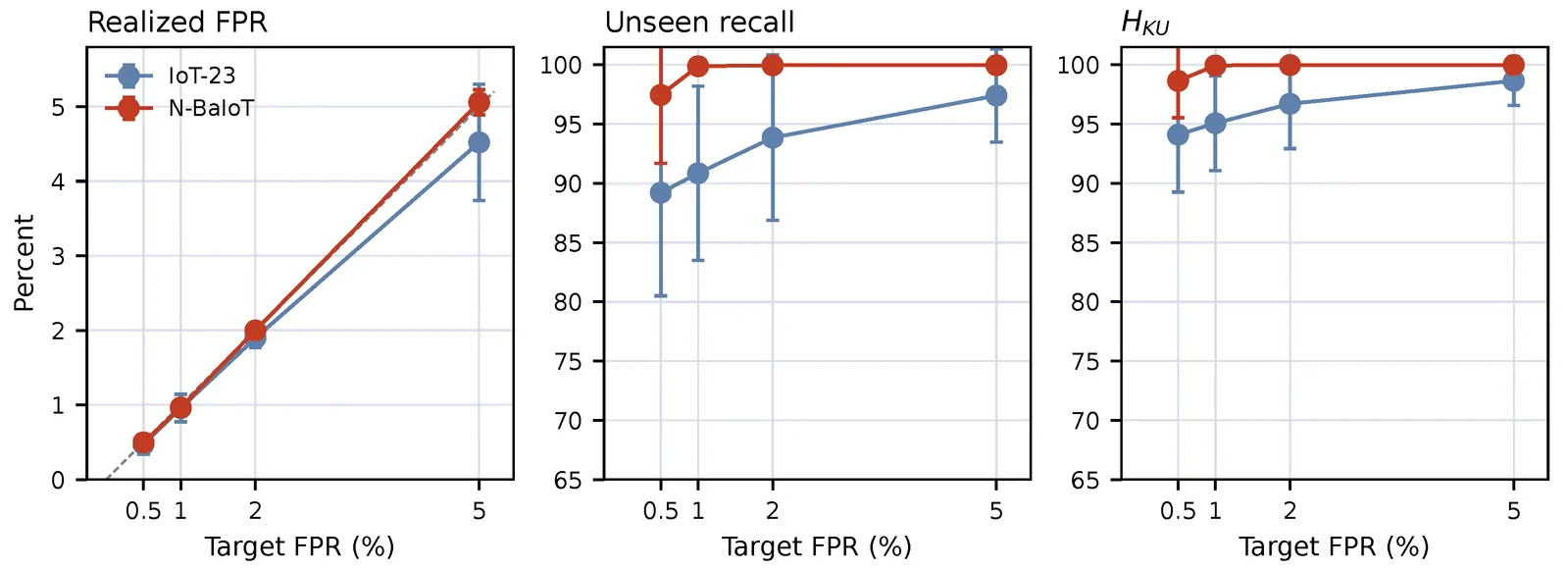
Operating-point sensitivity of budgeted MCDE. Points show macro means over the main held-out families, and error bars show one standard deviation. Realized FPR is measured on the held-out benign test set; the dashed line denotes equality between requested and realized FPR.

For a fitted detector, the operating point is determined by the final decision threshold and specifies the tolerated false-alarm rate on benign traffic. This setting is important in IoT monitoring because different deployments may impose different false-alarm budgets. We therefore examine whether MCDE can adapt to several requested FPR levels through threshold recalibration alone, while preserving the fitted feature transformation, benign modes, evidence references, and detector parameters. With K=15 and diagonal Mahalanobis distance, we recalibrate the final threshold for target FPRs of 0.5%, 1%, 2%, and 5%. Each point in Figure 6 summarizes nine IoT-23 or six N-BaIoT family–seed runs.

Figure 6 shows that the macro-mean benign FPR closely follows the requested level on both datasets. Relaxing the target FPR increases unseen recall and $H_{KU}$, with the largest gain on N-BaIoT between 0.5% and 1%, while IoT-23 shows more gradual improvement. These results illustrate the expected false-alarm/detection trade-off and show that the operating point can be adjusted without retraining. We retain the 1% operating point as a common low-FPR setting, not as a threshold selected to maximize recall.

### 4.8. Computational Efficiency

**Figure 7:**
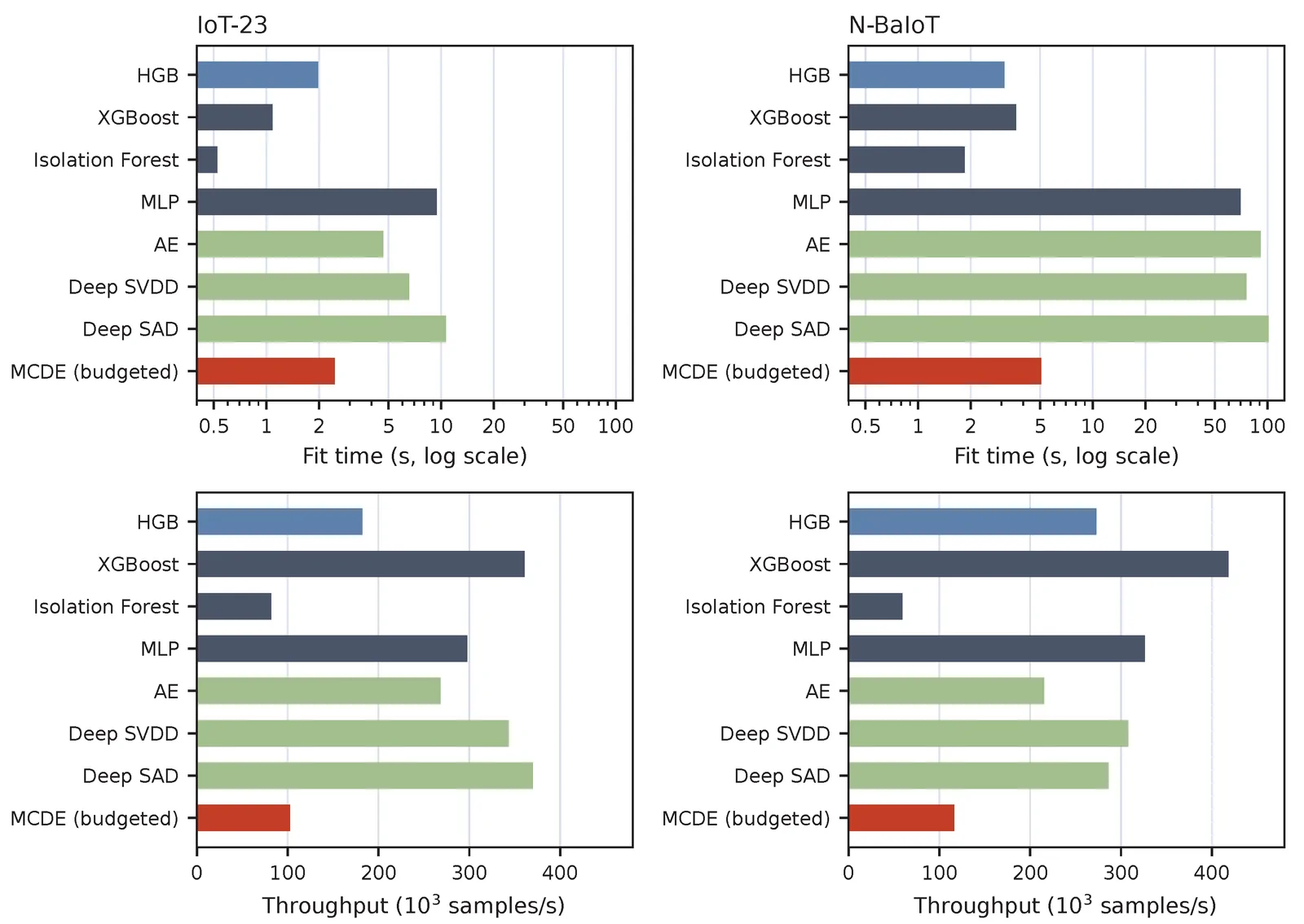
Visualization of the model fit (s) and throughput (k/s) metrics.

This experiment evaluates the practical cost of MCDE against the seven baselines under isolated CPU-only execution with eight threads. All methods use the architectures and hyperparameters from the effectiveness experiments; IoT-23 holds out DDoS and N-BaIoT holds out Gafgyt, both with seed 42. We report core model-fitting time, inference throughput (median over five repetitions on a 50,000-sample workload, including preprocessing and scoring), peak incremental RSS, and deployment-bundle size. Figure 7 visualizes fitting time and throughput; Table 11 reports the complete measurements.

As shown in Table 11, MCDE requires only 2.46 s and 5.08 s for core model fitting on IoT-23 and N-BaIoT, respectively, corresponding to approximately 1.9× and 13.8× speedups over the fastest neural baseline without requiring an accelerator. Its complete deployment bundles are also compact, at 0.695 and 1.108 MiB. Figure 7 shows that this model-fitting advantage does not extend to inference throughput. MCDE is slower than most tree and neural baselines, although it still processes more than 100,000 samples per second on both datasets. Its process-level peak RSS is also relatively high, so MCDE should not be interpreted as memory-minimal. Therefore, its practical advantage lies in short CPU-only model fitting, accelerator-free operation, compact deployment storage, and sufficient inference throughput, making it suitable for network gateways or monitoring systems rather than direct execution on resource-constrained IoT endpoints.

## 5. Conclusions

This paper presented MCDE for IoT traffic anomaly detection when attack-family knowledge is incomplete. MCDE combines supervised known-attack evidence with multi-mode benign-deviation evidence and maps their heterogeneous outputs to a common empirical benign-tail scale. It then incorporates branch-wise shares of the target FPR into a budget-normalized fusion rule and calibrates the decision threshold on a disjoint held-out benign set. The fusion also admits an interpretation through budget-adjusted benign-tail self-information. Under valid branch-tail probabilities, the derived union-bound result limits the combined nominal benign-alarm probability without requiring branch independence.

Comprehensive experiments on IoT-23 and N-BaIoT support the effectiveness of MCDE under the evaluated low-FPR family-hold-out conditions. At the 1% target FPR, MCDE achieves 90.85% unseen recall, 100% known recall, and 95.07% $H_{KU}$ on the three principal IoT-23 family hold-outs, with a benign-test FPR of 0.96%. It also preserves near-ceiling performance on N-BaIoT, achieving 99.87% unseen recall and 99.94% $H_{KU}$. The complementary hold-out and stress tests further support its effectiveness under different unseen-attack conditions, while the ablation results verify the complementary roles of the two evidence branches and the importance of budgeted fusion. Sensitivity and CPU-only efficiency experiments additionally indicate a stable parameter region, short fitting times, compact deployment bundles, and throughput suitable for gateway-side monitoring.

However, these findings and the associated FPR-control results should be interpreted within the stated calibration conditions and evaluation scope. The nominal result requires valid benign-tail probabilities, including for any pooled-fallback mapping, while final-threshold calibration directly bounds only the empirical FPR on the calibration set. Extending this control to future traffic requires compatibility between deployment and calibration distributions. The benign-device transfer results show that distribution changes can still concentrate false alarms. Therefore, MCDE does not establish unconditional robustness under cross-network or temporal shifts. Future work will investigate broader temporal and cross-network evaluation together with trusted updates of empirical references or benign modes to improve FPR stability in changing deployment environments.

## Figures and Tables

**Table 1:** Summary of IoT traffic anomaly detection approaches according to the attack information available during model development.

Detection Route and Representative Studies	Information Used During Development	Capability and Limitation
Detection from labeled known attacks [10,11,16,23,24]	Labeled benign and known-attack samples.	Strong known-family discrimination; unseen recall depends on the learned attack coverage.
Detection with limited or incrementally acquired attack information [17,18,26,27,28]	Few target labels, target-domain data, feedback, or model updates.	Can adapt as target information arrives; requires information unavailable in static hold-out detection.
Detection without labeled attack information [7,12,14,15,20,21]	Benign reference traffic or unlabeled traffic with rare anomalies.	Detects deviation without attack labels; does not directly use known-attack discrimination and may miss near-benign attacks.

**Table 2:** Summary of the public datasets and their representations in this study.

Dataset	Original Scale	Benign Source	Attack Data Used	Input Representation	Role in This Study
N-BaIoT	7,062,606 records from nine commercial devices	Benign traffic from the same nine devices	Gafgyt and Mirai; five attack types per family	115 numerical multi-window traffic statistics	Two malware-family hold-outs and ten attack-type hold-outs
IoT-23	23 scenarios: 20 malicious and three benign	Three IoT devices in benign scenarios	DDoS, horizontal port scanning, C&C, and rare-family Okiru	16 Zeek connection fields: 10 numerical and six categorical	Three main family hold-outs and one rare-family stress test
CICIoT2023	105 IoT devices, 33 attack types	Benign traffic from the same testbed	DDoS, DoS, Mirai	39 flow-level features	Cross-dataset validation with three family hold-outs

**Table 3:** Realized benign split sizes and attack-sample caps used by the primary family-hold-out runs. “Per known unit” denotes an IoT-23 family or an N-BaIoT attack type.

Dataset	Benign Train	$B_{c1}$	$B_{c2}$	Benign Test	Known Train/Test (Each)	Unseen Test
N-BaIoT	150,000	∼29,000	∼29,000	50,000	15k/type	50,000
IoT-23	∼22,600	∼15,000	∼6300	∼6300	25k/fam.	≤50,000

**Table 4:** Evaluation metrics used in the experiments. Primary metrics are used for the main method comparison; diagnostic metrics characterize ranking and error concentration.

Metric	Definition	Purpose
Requested FPR α	Preset operating target, primarily 1%	Specifies the false-alarm operating point before evaluation.
Calibration FPR	$\frac{1}{|B_{c2}|}\sum_{x\in B_{c2}}I\left[S\left(x\right)>\tau_{\alpha }\right]$	Ensures the threshold respects the target FPR on the disjoint calibration set.
Normal FPR	$\frac{1}{N_{B}}\sum_{x\in B}I\left[S\left(x\right)>\tau_{\alpha }\right]$	Test-time false-alarm rate on benign traffic.
Known recall $R_{K}$	$\frac{1}{N_{K}}\sum_{x\in D_{K}}I\left[S\left(x\right)>\tau_{\alpha }\right]$	Detection rate for attack families represented in training.
Unseen recall $R_{U}$	$\frac{1}{N_{U}}\sum_{x\in D_{U}}I\left[S\left(x\right)>\tau_{\alpha }\right]$	Detection rate for attack families absent from development.
Harmonic known–unseen recall $H_{KU}$	$\frac{2R_{K}R_{U}}{R_{K}+R_{U}}$	Balanced primary metric; penalizes sacrificing either recall.
Unseen AUROC	AUROC on $B\cup D_{U}$	Threshold-independent ranking quality for benign vs. unseen attacks.
Unseen AUPRC	Area under precision–recall curve on $B\cup D_{U}$	Ranking measure sensitive to class proportions.
Worst supported-group FPR	$\max_{g:n_{g}\geq 100}{\mathrm{FPR}}_{g}$	Primary group-wise FPR measure; excludes groups with <100 benign test samples.
Group-FPR standard deviation	$\mathrm{std}{\left\{{\mathrm{FPR}}_{g}\right\}}_{g}$	Measures unevenness of false alarms across groups.

**Table 5:** IoT-23 unseen-family results at a target benign FPR of 1%. Values are mean ± standard deviation over three held-out families and three seeds. Higher is better for recall and AUPRC; lower is better for FPR.

Method	Normal FPR (%)	$R_{K}$ (%)	$R_{U}$ (%)	$H_{\mathrm{KU}}$ (%)	Unseen AUPRC (%)	Worst Supported-Capture FPR (%)
HGB	0.918 ± 0.226	100.000 ± 0.001	85.768 ± 13.483	91.823 ± 8.007	99.259 ± 1.065	2.867 ± 1.064
XGBoost	0.943 ± 0.159	99.999 ± 0.001	85.125 ± 14.304	91.377 ± 8.572	99.290 ± 1.018	2.830 ± 0.599
MLP	0.980 ± 0.151	99.988 ± 0.020	66.024 ± 39.748	71.011 ± 40.426	95.006 ± 7.543	20.484 ± 14.641
Isolation Forest	1.010 ± 0.094	78.732 ± 15.772	78.824 ± 27.452	73.371 ± 19.555	99.197 ± 0.691	5.650 ± 0.489
AE	1.054 ± 0.112	87.998 ± 6.578	87.032 ± 12.366	86.678 ± 4.126	99.477 ± 0.468	5.838 ± 0.747
Deep SVDD	1.092 ± 0.117	92.294 ± 4.293	91.305 ± 8.488	91.408 ± 2.568	99.675 ± 0.381	4.774 ± 1.571
Deep SAD	1.021 ± 0.113	99.998 ± 0.004	90.252 ± 12.405	94.459 ± 7.196	99.703 ± 0.412	7.854 ± 8.450
**MCDE**	0.959 ± 0.185	100.000 ± 0.001	90.846 ± 7.355	95.066 ± 3.990	99.931 ± 0.074	2.622 ± 0.686

Note: The proposed method name, MCDE, is shown in bold for identification. This convention also applies to subsequent tables.

**Table 6:** N-BaIoT unseen-family results at a target benign FPR of 1%. Values are mean ± standard deviation over two held-out families and three seeds. Higher is better for recall and AUPRC; lower is better for FPR.

Method	Normal FPR (%)	$R_{K}$ (%)	$R_{U}$ (%)	$H_{\mathrm{KU}}$ (%)	Unseen AUPRC (%)	Worst-Device FPR (%)
HGB	0.982 ± 0.063	100.000 ± 0.000	99.886 ± 0.126	99.943 ± 0.063	99.846 ± 0.133	2.725 ± 0.376
XGBoost	1.006 ± 0.081	100.000 ± 0.000	99.859 ± 0.155	99.929 ± 0.078	99.981 ± 0.012	2.621 ± 0.202
MLP	0.986 ± 0.047	99.998 ± 0.003	99.587 ± 0.424	99.792 ± 0.214	99.946 ± 0.019	1.935 ± 0.282
Isolation Forest	0.941 ± 0.026	94.071 ± 8.893	95.808 ± 6.189	94.541 ± 4.614	99.524 ± 0.208	2.701 ± 0.089
AE	0.970 ± 0.071	87.624 ± 12.023	88.993 ± 11.798	87.859 ± 10.557	99.483 ± 0.414	3.589 ± 0.577
Deep SVDD	0.948 ± 0.067	84.505 ± 13.677	93.998 ± 8.704	88.877 ± 11.458	99.367 ± 0.602	3.106 ± 0.456
Deep SAD	0.980 ± 0.072	100.000 ± 0.001	99.869 ± 0.144	99.934 ± 0.072	99.992 ± 0.010	2.234 ± 0.437
**MCDE**	0.965 ± 0.076	100.000 ± 0.000	99.874 ± 0.139	99.937 ± 0.069	99.866 ± 0.113	2.505 ± 0.564

**Table 7:** CIC IoT 2023 family-hold-out stress-test results at the 1% target-FPR operating point.

Held-Out Family	Benign FPR (%)	Known Recall (%)	Unseen Recall (%)	$H_{KU}$ (%)	Unseen AUROC	Unseen AUPRC	Worst Supported FPR (%)
DDoS	0.978 ± 0.061	78.729 ± 0.280	99.996 ± 0.004	88.097 ± 0.177	0.999970 ± 0.000008	0.999952 ± 0.000032	2.132 ± 0.195
DoS	0.910 ± 0.101	87.508 ± 0.184	99.998 ± 0.000	93.337 ± 0.105	0.999978 ± 0.000019	0.999957 ± 0.000038	1.989 ± 0.303
Mirai	0.934 ± 0.082	88.193 ± 0.146	99.998 ± 0.002	93.725 ± 0.083	0.999994 ± 0.000007	0.999989 ± 0.000016	2.035 ± 0.242
Macro	0.940 ± 0.078	84.810 ± 4.574	99.997 ± 0.002	91.720 ± 2.725	0.999981 ± 0.000015	0.999966 ± 0.000031	2.052 ± 0.226

**Table 8:** IoT-23 ablation results at the 1% target-FPR operating point. Values are mean ± standard deviation. The three-family result contains nine family–seed runs, the Okiru result contains three runs, and the four-condition result contains all twelve runs.

Variant	Benign Evidence	HGB	Fusion	$H_{KU}$ (%)		
				Main Families	Okiru	Four Conditions
V1	Global distance	No	–	89.051 ± 4.429	68.189 ± 47.679	83.836 ± 22.729
V2	Mode distance	No	–	92.940 ± 2.322	15.264 ± 3.264	73.521 ± 35.213
V3	None	Yes	–	91.823 ± 8.007	99.449 ± 0.954	93.729 ± 7.660
V4	Global-tail evidence	Yes	Maximum	93.005 ± 7.279	100.000 ± 0.000	94.754 ± 6.967
V5	Mode-tail evidence	Yes	Mean	93.917 ± 8.039	71.198 ± 49.850	88.237 ± 24.585
V6	Mode-tail evidence	Yes	Maximum	95.671 ± 3.499	13.401 ± 0.204	75.103 ± 37.328
**MCDE**	Mode-tail evidence	Yes	FPR-budgeted	95.066 ± 3.990	99.449 ± 0.954	96.162 ± 3.959

**Table 9:** Sensitivity to the supervised FPR-budget share at the 1% target FPR. IoT-23 values are macro means over the three principal family hold-outs and three seeds. Okiru values are means over its three-seed low-diversity stress test.

*w*	IoT-23 $R_{U}$ (%)	IoT-23 $H_{KU}$ (%)	Okiru $R_{U}$ (%)	Okiru $H_{KU}$ (%)
0.10	92.97	96.27	2.98	6.35
0.20	92.96	96.26	4.15	6.87
0.30	92.96	96.27	4.21	8.41
0.50	91.89	95.67	4.95	9.39
0.70	90.64	94.96	35.50	38.09
0.80	90.55	94.91	68.70	70.50
0.90	90.85	95.07	98.92	99.45
0.95	90.49	94.84	98.92	99.45
0.99	85.77	91.82	98.92	99.45

**Table 10:** Distance-metric sensitivity for budgeted MCDE at K=15 and a 1% target benign FPR. Values are seed-42 macro means over three IoT-23 families or two N-BaIoT family hold-outs.

Dataset	Distance	$R_{U}$ (%)	Unseen AUROC (%)	Unseen AUPRC (%)
IoT-23	RMS Euclidean	88.07	95.82	99.34
	Diagonal Mahalanobis	90.86	99.63	99.95
N-BaIoT	RMS Euclidean	99.87	99.83	99.82
	Diagonal Mahalanobis	99.88	99.88	99.85

**Table 11:** Fair CPU-only computational comparison. Model fit excludes preprocessing and calibration; throughput includes preprocessing and scoring of 50,000 samples; ΔRSS is incremental peak process memory over the complete benchmark worker.

Dataset	Method	Model Fit (s)	Throughput (k/s)	μs/Sample	ΔRSS (MiB)	Bundle (MiB)
IoT-23	HGB	1.97	182.17	5.49	409.7	0.539
	XGBoost	1.08	360.77	2.77	314.7	0.368
	Isolation Forest	0.52	81.60	12.25	299.1	24.501
	MLP	9.45	297.68	3.36	403.6	0.218
	AE	4.66	268.46	3.72	368.6	0.161
	Deep SVDD	6.57	343.34	2.91	369.6	0.084
	Deep SAD	10.68	369.95	2.70	369.4	0.084
	MCDE	2.46	102.42	9.76	406.2	0.695
N-BaIoT	HGB	3.13	272.89	3.66	973.0	0.654
	XGBoost	3.64	418.44	2.39	644.6	0.295
	Isolation Forest	1.85	59.22	16.89	744.4	106.444
	MLP	70.25	326.05	3.07	744.0	0.219
	AE	91.44	215.45	4.64	645.3	0.445
	Deep SVDD	75.76	307.86	3.25	744.1	0.228
	Deep SAD	101.27	286.05	3.50	645.6	0.228
	MCDE	5.08	116.40	8.59	970.9	1.108

## Data Availability

The N-BaIoT dataset is publicly available from the UCI Machine Learning Repository at https://archive.ics.uci.edu/dataset/442/detection+of+iot+botnet+attacks+n+baiot, and the IoT-23 dataset is publicly available from the Stratosphere Laboratory at https://www.stratosphereips.org/datasets-iot23 (both accessed on 3 August 2026) [7,35]. The implementation code and generated experimental results will be made publicly available upon publication; before publication, they are available from the corresponding author upon reasonable request.

## Abbreviations

The following abbreviations are used in this manuscript:

IoT	Internet of Things
MCDE	Mode-Calibrated Dual-Evidence Detector
FPR	False-Positive Rate
AUROC	Area Under the Receiver Operating Characteristic Curve
AUPRC	Area Under the Precision–Recall Curve
HGB	HistGradientBoosting
AE	Autoencoder
